# Personalized, Evidence-Informed Training Plans and Exercise Prescriptions for Performance, Fitness and Health

**DOI:** 10.1007/s40279-021-01495-w

**Published:** 2021-06-18

**Authors:** Henning Wackerhage, Brad J. Schoenfeld

**Affiliations:** 1 Exercise Biology Group, Department of Sport and Health Sciences, Technical University of Munich, Georg-Brauchle-Ring 60/62, 80992 Munich, Germany; 2 Department of Health Sciences, Lehman College, Bronx, NY USA

## Abstract

A training plan, or an exercise prescription, is the point where we translate sport and exercise science into practice. As in medicine, good practice requires writing a training plan or prescribing an exercise programme based on the best current scientific evidence. A key issue, however, is that a training plan or exercise prescription is typically a mix of many interacting interventions (e.g. exercises and nutritional recommendations) that additionally change over time due to periodisation or tapering. Thus, it is virtually impossible to base a complex long-term training plan fully on scientific evidence. We, therefore, speak of evidence-informed training plans and exercise prescriptions to highlight that only some of the underlying decisions are made using an evidence-based decision approach. Another challenge is that the adaptation to a given, e.g. endurance or resistance training programme is often highly variable. Until biomarkers for trainability are identified, we must therefore continue to test athletes, clients, or patients, and monitor training variables via a training log to determine whether an individual sufficiently responds to a training intervention or else re-plan. Based on these ideas, we propose a subjective, pragmatic six-step approach that details how to write a training plan or exercise prescription that is partially based on scientific evidence. Finally, we advocate an athlete, client and patient-centered approach whereby an individual’s needs and abilities are the main consideration behind all decision-making. This implies that sometimes the most effective form of training is eschewed if the athlete, client or patient has other wishes.

## Key Points

**Table:** 

A training plan is a complex mix of many interventions that often change over time due to periodization. In contrast to a “drug or no drug” decision in medicine, it is virtually impossible to base all of the underlying decisions on scientific evidence.
An alternative to a fully evidence-based approach is an evidence-informed approach where only some training plan decisions are based on the currently best available evidence.
Many training adaptations vary greatly. Until robust trainability biomarkers are identified, practitioners should continue to test athletes, clients, or patients and monitor training variables to re-plan if a training prescription does not yield the desired adaptations.

## Introduction to Evidence-Based Practice

Physicians, physiotherapists, lifestyle and sports coaches, as well as fitness trainers all have one thing in common: they prescribe interventions to change the human body. The aims of physicians are to prevent, treat or ideally cure diseases by prescribing medical treatments. In contrast, sports coaches and fitness trainers aim to improve athletic performance, fitness and/or health by writing training plans or prescribing exercise often combined with nutritional recommendations. In doing so, physicians and sport practitioners can choose from a myriad of possible interventions including resistance or endurance exercise training, a Mediterranean, vegan or protein-rich diet or, in the case of medicine, drugs or surgery. But how can one select those interventions that most likely to achieve the desired treatment effects or adaptations in an individual?

For many years, practitioners simply followed their opinion or the opinion of experts published in books or on the internet plus perhaps information from some random scientific papers. For example, “experts” have recommended that endurance can be best improved by training at the lactate threshold or that sets with 8–12 repetition maximum are “optimal” for muscle hypertrophy, despite a paucity of research supporting these beliefs. In medicine, however, the advent of evidence-based medicine has changed medical decision-making [1] and this approach is also now used by other disciplines as “evidence-based practice”.

The aim of this current opinion article is to introduce evidence-based and personalized practice to sport and exercise practitioners. Specifically, we will

1. Discuss the history of evidence-based medicine and practice;
2. Provide a subjective, “how-to”, six-step guideline on how to develop an evidence-informed training plan or exercise prescription;
3. Recommend strategies to account for inter-individual variation, especially in trainability.

## History of Evidence-Based Practice

Historically, physicians selected treatments based on opinion, religious belief or ill-defined experience. This included the use of bloodletting for various ailments [2] and mercury for syphilis [3], which are not only ineffective treatments, but detrimental. Over time, medical researchers started to carry out intervention studies, which are studies that aim to compare the effectiveness of an intervention either to no treatment or to the current best treatment. Perhaps the earliest intervention study in sport and exercise science was reported by Ben Cao Tu Jing in the eleventh century:

“*It was said that in order to evaluate the effect of genuine Shangdang ginseng, two persons were asked to run together. One was given the ginseng while the other ran without. After running for approximately three to five li* [≈1500 to 2500 m], *the one without the ginseng developed severe shortness of breath, while the one who took the ginseng breathed evenly and smoothly*” [4].

Whilst this intervention study provided evidence for beneficial effects of ginseng, the evidence is low quality: the breathing measurement is subjective, the subjects probably differed at baseline, there was only one subject per group and the researcher probably had a conflict of interest as he seemed enthusiastic about ginseng. This highlights that scientific evidence is not automatically a truth but needs to be interpreted critically. Over time, scientists learned to design more robust intervention experiments as researchers improved scientific methods and reduced bias, and other sources of error.

Since Ben Cao Tu Jing’s historical experiment, researchers have used many study types to investigate the effect of a given medical treatment or exercise on an outcome. The most important difference is the use of observational studies, where the investigator just observes the effect of a treatment but does not control the treatment, versus experimental studies where the experimenter administers the treatment or the intervention. A key step forward was the advent of randomized control trials (RCTs) with additional control measures such as blinding and placebos as advocated by Archie Cochrane, who deemed these designs a superior alternative to observational studies and anecdotal opinions [5].

So how has the refinement of studies that test the effectiveness of an intervention led to “evidence-based practice”? The actual term “evidence-based medicine”, or more broadly “evidence-based practice”, was first used in the 1990s by researchers at McMaster University and has been especially linked to David Sackett who, from 1994 to 1999, led the Centre for evidence-based Medicine at the University of Oxford. In a review titled “Evidence based medicine: what it is and what it isn’t,” Sackett and colleagues define evidence-based medicine as …

“*the conscientious, explicit, and judicious use of the current best evidence in making decisions about the care of individual patients*” [1].

By that definition, David Sackett and colleagues instruct evidence-based practitioners to do two things: First, gather all of the currently available scientific evidence of sufficient quality and second, interpret and apply that evidence. Importantly, the terms, “*conscientious, explicit, and judicious use*” mean that we do not necessarily need to do exactly what the evidence says; rather, we have the option to make alternative, subjective decisions if we feel that there is a good reason to do so.

Several issues must be considered when judging the available evidence. First, not all scientific data is of equal quality. Single pieces of evidence include subjective “expert” opinion, case reports and series, non-randomised control trials, and randomised control trials. On the path towards becoming an evidence-based practitioner, we must, therefore, develop the skill of critically interpreting scientific evidence. To aid this, e.g. Greenhalgh has published book “How to read a paper” [6].

**Fig. 1:**
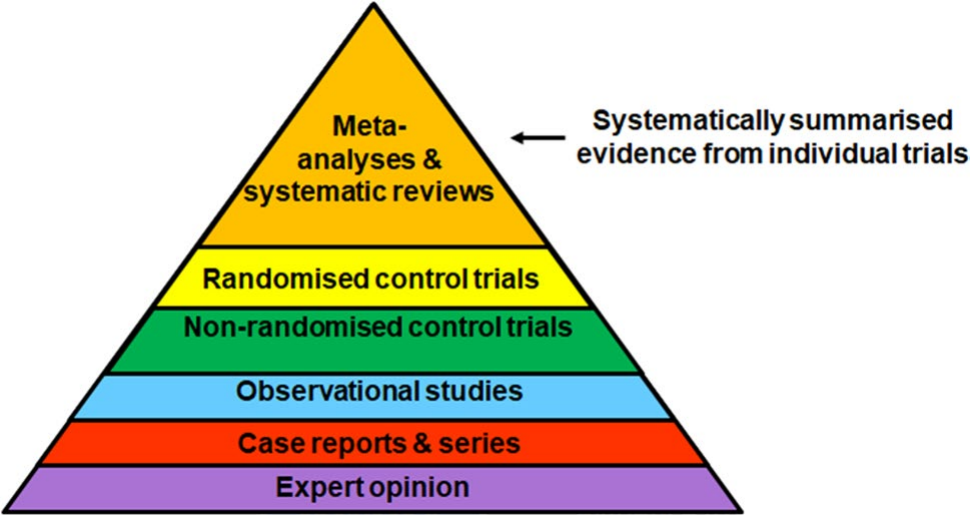
Evidence pyramid. The higher the form of evidence, the more robust is the evidence in most cases. However, whilst study type is a key factor that determines the quality of the evidence, it is not the only criterion and so also randomized control trial data need to also be critically analysed [7]

A second issue is that it is impractical and not time-effective to systematically search for and read all the *current best evidence* for an intervention decision as there may be dozens of observational or experimental studies in relation to a single intervention such as the use of aspirin for treating headaches. This demand has led to systematic reviews and meta-analyses as two new publication types that support evidence-based practice as they aim to synthesize the current best evidence for treatment decisions. These forms of evidence also comprise the tip of the so-called evidence pyramid (Fig. 1), which illustrates the hierarchy for different levels of evidence [7]. As displayed in the hierarchy, expert opinion ranks as the lowest form of evidence, randomized control trials rank as the highest evidence for a single trial, and systematic reviews and meta-analyses rank as the highest form of overall evidence, as these studies attempt to synthesize the scientific evidence for a treatment decision or other topic.

Importantly, the conclusion of the evidence pyramid is not that less robust study types are flawed, should not be carried out and be ignored as evidence for training decisions. For example, case studies or case series are an essential study type to generate scientific evidence if it is impossible to recruit sufficient numbers of subjects or if the interventions are too long for well-controlled trials. Examples are case studies of world-class athletes or astronauts, or when an athlete is studied, e.g. over a full 4 year Olympic cycle.

The quality of an evidence statement can be graded using different options including the detailed grading system by the Oxford Centre for Evidence-Based Medicine [8]. A much simpler grading system is the GRADE (short for Grading of Recommendations Assessment, Development and Evaluation) system [9], which has four levels of evidence in relation to the effect of an intervention such as exercise training:

- *Very low quality* Any estimate of effect is very uncertain.
- *Low quality* Further research is very likely to have an important impact on our confidence in the estimate of effect and is likely to change the estimate.
- *Moderate quality* Further research is likely to have an important impact on our confidence in the estimate of effect and may change the estimate.
- *High quality* Further research is unlikely to change our confidence in the estimate of effect.

Grading the quality of evidence is good practice as it indicates the confidence that can be placed on its veracity. Importantly, a “very low quality” rating does not mean that, e.g. a given exercise training intervention does not cause an adaptation; it just means there is a lack of supporting scientific evidence for its use or, put simply, we do not know whether it works. In contrast, if the evidence for a training intervention is rated “high quality”, then we can expect it likely causes the desired effect. Caveats are that sometimes the effects differ across populations, and that the evidence only refers to the average response which is problematic if there is a large variation in trainability (as is common in applied exercise interventions).

Evidence-based medicine has now become the gold standard in medical decision-making, i.e. in deciding how to treat a patient. That said, evidence-based medicine is not without issues, as summarized by Greenhalgh et al. [10]. One such problem is that conflicts of interest can lead to the design of biased trials, the non-reporting of negative results, and/or the biased reporting of data [11]. This is of particular concern for company-funded medical and exercise intervention trials. For example, if Sports Drink A had a high amount of sugar, then a biased randomized control trial design could potentially use glycogen-depleted individuals and then test whether subjects run a faster marathon whilst drinking the sports drink versus water. Because of the importance of sufficient glycogen stores and of carbohydrate ingestion for marathon running [12], we would expect that the biased design of the experiment will yield the desired results; namely, fasted subjects will probably run the marathon faster when consuming the sugary sports drink compared to water.

A second problem is that statistical analyses often employed in the applied sciences are inherently flawed from a decision-making standpoint. A primary problem here is the focus on null hypothesis statistical testing (i.e. *p* values < 0.05 used for accept-or-reject-hypothesis conclusions), which has been termed “dichotomania”. The issue is that we should never conclude ‘no difference’ or ‘no relationship’ in an intervention simply because a *p* value is above a given threshold of “statistical significance” [13]. Instead, *p* values simply indicate how probable an effect is and should be considered just one of several decision-making criterions. It can be argued that magnitude-based statistics such as effect sizes and confidence intervals are potentially more valuable for deciding whether, e.g. the adaptation to a training intervention is likely more than just measurement variability and biologically meaningful [13, 14]. Moreover, when interpreting research findings, one should also look at individual effects via graphs that present data points for each participant, as there may be responders and non-responders [15–18]. We know for example, that the adaptation to the same endurance [19, 20] or resistance training [21, 22] varies greatly and so, e.g. a training might trigger the desired adaptation only in some individuals but not in others.

A third point is the research-based focus on hypotheses because a hypothesis is essentially a subjective statement of the outcome of an experiment before the actual experiment is conducted and so arguably, a hypothesis introduces bias. Despite these obvious flaws and even though few current papers in esteemed journals such as *Nature*, *Science* or *Cell* appear to state hypotheses, they have nonetheless turned into a dogma of scientific theory. David Glass has questioned the use of hypotheses and Popper’s falsification idea; instead of hypotheses, he recommends open research questions, as open questions are not only less biased but also more intuitive than hypotheses [23]. This is a controversial point.

A fourth issue is that the volume of evidence or specifically the number of publications is large for many treatment decisions [10]. For example, a Pubmed search for “exercise” and “training” revealed 461,115 publications as of May 2021, which are beyond the reading capacity of a sole individual. However, systematic searches for specific keywords can generally reduce the number of publications to a manageable level. In addition, systematic reviews and meta-analyses can help to synthesize the results of many studies related to specific interventions. There are caveats, however, as meta-analyses may pool data from studies of variable study quality. Consequently, conclusions from such analyses are only as good as the quality of the included individual studies. Thus, evidence-based practitioners should avoid treating systematic reviews and meta-analyses as scientific truths. Rather, it is essential to critically assess whether they have been well-conducted and whether the population, intervention, controls and outcomes are similar as in the planned intervention about which we wish to learn.

## Evidence-Based Practice and Personalization

When judging evidence, practitioners often focus on the mean effect of an intervention even though there might be adverse responders, non-responders, average responders and extreme responders to that intervention. Current medicine has recognized this problem and now aims to develop strategies. This type of medicine has been termed personalized, individualized or precision medicine [24]. Highly variable responses to treatment are, however, not limited to medicine. Similarly the changes in V̇O_2_max [19], risk factors after endurance training [20], and strength and muscle mass after resistance training [21, 22] all vary widely from person to person and some individuals appear to worsen their V̇O_2_max, strength and risk factors after exercise training.

A key aim in decision-making is to avoid putting individuals on a lengthy, costly or painful treatment or on an, e.g. 6-month exercise intervention only to find out that the intervention was ineffective or has caused harm. Accordingly, personalized medicine or practice seeks to measure biomarkers such as DNA sequence variants or circulating molecules that inform as to whether an individual will likely respond to a treatment or not. However, despite intense efforts to identify measurable exercise-related biomarkers for, e.g. the response of the V̇O_2_max to endurance training [25, 26], no robust set of measurable biomarkers has emerged that can reliably be used to decide e.g. whether or not a client will respond to endurance training with a sufficiently large increase of the V̇O_2_max. Thus, the pragmatic approach to variation of trainability is to measure the key variables that a training programme should improve repeatedly, and determine whether an individual responds sufficiently to exercise training as is common practice. If an athlete, client or patient does not respond then practitioners should look for a plan B which could be to simply increase the dose of exercise [27]. Research on the topic is ongoing. For example, one aim of the current MoTrPac project that is funded with $170 million in the USA is to identify biomarkers for the magnitude of adaptation to endurance or resistance exercise [28]. These efforts will hopefully provide practitioners with tools to help personalize training prescription in the future.

## Three Examples of Evidence-Based Controversies in Sport and Exercise Science

To further make a case for evidence-based practice, we will now discuss sport and exercise-related recommendations where there are issues with scientific evidence.

## Stretching to Prevent Injuries

Many exercisers stretch before or after the main part of an exercise session, commonly as a strategy to avoid sports injuries. But is there any evidence for this belief? For nearly 20 years investigators have meta-analysed data on stretching and injury risk; the combined results of these analyses indicate that stretching does not meaningfully reduce the risk of injury [29–31]. One meta-analysis even compared several exercise interventions to prevent sports injuries and concluded that all the included injury prevention strategies worked *except* for stretching [32]! Thus, in summary, there is no scientific evidence for the commonly held belief that stretching can be used to prevent sports injuries. In fact, evidence indicates that stretching does not prevent injuries. This is, therefore, an example of a commonly held belief with little supporting evidence to back up its veracity.

## Block Periodization

Most athletes sub-divide their training year into training periods such as preparatory, competition and transition periods, a practice that has been termed periodization [33]. An alternative to the three periodization phases (preparatory, competitive & transition) is the so-called “block” periodization model where athletes focus on one adaptation such as strength or speed in one training block [34–36]. In his 2019 review, Issurin used a semi-systematic review strategy to analyse the biological mechanisms of block periodization and to compare the model to a traditional periodization model, primarily specific to training prescription for endurance sports [34]. On the surface, phrases such as “*intense transmutation block mesocycles* “, “*accumulation, transformation and realization blocks*” or “*secretion of growth-related genes and fibroblast growth factor-inducible 14*” suggest a deep understanding of molecular biology whereas the compilation of training studies in a table suggests a detailed analysis of block training studies versus controls [34]. However, when scrutinizing the claims more closely it becomes clear that the molecular data are random observations that do not necessarily support the claim that block periodization is superior to other types of training. Moreover, the cited intervention studies do not compare block periodization to a matched control intervention, limiting the ability to adequately compare findings across models. These flaws were highlighted in a commentary that argued Issurin accepted favourable findings more readily than unfavourable findings, termed confirmation bias, used superficially rational chains of reasoning and overoptimistic statements such as “[block periodization] *has found strong support in molecular biology*” for which there is little evidence [37]. In summary, the evidence presented by Issurin in his review [34] is scant evidence indicating that block periodization is a superior form of periodisation per se. The absence of evidence for block periodisation, however, does not mean that block periodization does not work, or that it may not be superior to other periodization models. The issues in relation to block periodisation are unfortunate because many well trained and successful elite athletes appear to employ block periodization in their training programs. For this reason, block periodization should not be dismissed prematurely [38].

## ACSM Position Stands on Resistance Training

In 2002, the American College of Sports Medicine published a position stand on resistance training for novice, intermediate and advanced exercisers. The position stand makes specific recommendations about how many repetitions, sets and intensities are necessary to trigger certain adaptations [39]. This position stand was criticized by Carpinelli and colleagues, who claimed several of the studies cited in the paper do not support the claims and recommendations of the position stand [40]. The ACSM subsequently published an updated position stand in 2009, where it employed an A–D evidence category rating scale. In this position stand, the authors stated claims such as “*Training with loads of 60–70% of 1 RM for 8–12 repetitions for novice to intermediate individuals and cycling loads of 80–100% of 1 RM for advanced individuals. A.*” In this sentence, the “A” evidence rating *stands for “Randomized control trials (RCT; rich body of data)”* [41]. This new position stand was again criticized by Carpinelli, who concluded that “*the ACSM’s new Position Stand contains all the flaws that were pervasive in their previous Position Stand*” [42]. In summary, the ACSM position stand was written by respected scientists and practitioners who, whilst having considered and cited nearly 300 publications, may at times have misapplied the tools of evidence-based practice to justify their recommendations. Again, this does not mean that the recommendations are ineffective but, as with block periodization, we do not know whether there are better alternatives based on some of the evidence presented in the ACSM position stand.

In summary, these three examples show that there are sometimes issues with the evidence base for current training recommendations. This does not always imply that these training recommendations are ineffective or that future training recommendations must all be based on near-perfect evidence, as such evidence does often not exist. To us, the “lesson learned” is to honestly state the quality of the evidence and to avoid exaggerating evidence claims.

## Step-by-Step Workflow for Evidence-Informed Training Plans or Exercise Prescriptions

**Fig. 2:**
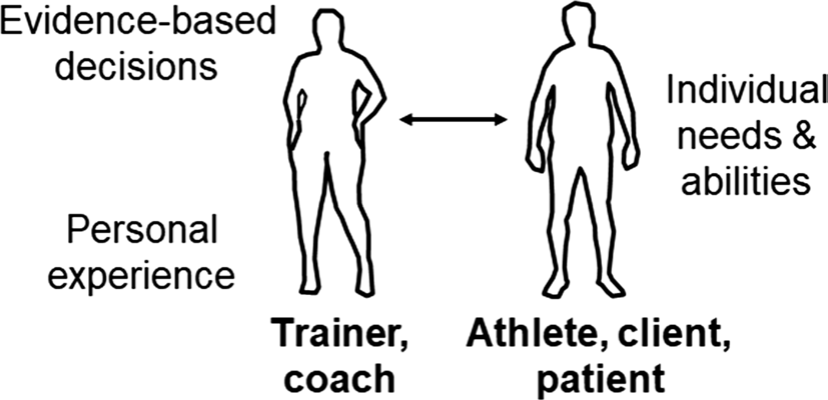
Interaction of a trainer or coach with an athlete, client or patient

In this section, we will now discuss how to apply evidence-based practice to writing a training plan or exercise prescription. When writing a training plan or prescribing exercise, trainers or coaches interact with athletes, clients or patients. In line with the concept of patient-centred care [43], we advocate athlete/client/patient-centred practice in most situations. This means that an individual’s performance, fitness or health needs and wishes are the driving force behind all decisions. Trainers and coaches then agree on goals, and write a training plan or prescribe exercise which is then discussed with the athlete, client or patient to see whether it fits their needs, wishes and abilities. This is illustrated in Fig. 2.

Exercise training interventions typically include a mix of many different exercises (e.g. 10 exercises for different muscles in a resistance training plan), with variable exercise intensities and durations (e.g. long slow runs and fast, intensive intervals in a marathon training plan). In addition, these interventions change over time due to periodization or tapering, and are combined with other interventions such as nutrition (e.g. a protein-rich diet or carbohydrate loading). Whilst it is possible to base some training plan decisions on the current best scientific evidence, it is virtually impossible in a complex, months-lasting training plan to base every decision on scientific evidence. Also, there will probably be no studies comparing the planned, complex intervention mix to a matched control intervention. For example, assume we aim to train a natural, female bodybuilder for 20 weeks, with 20 resistance exercises, nutritional and supplement recommendations, with a bulking and cutting period. Whilst we can easily find answers to questions such as “What are the general guidelines for maximizing muscle hypertrophy?” with scientific evidence [44, 45], we may rely mostly on subjective experience when choosing resistance exercises for each muscle. Also, it will be difficult to base the 20-week training intervention as a whole on scientific evidence as there will probably no publications comparing a 20-week training intervention in, e.g. 20 natural, female bodybuilders that resembles our training plan to 20 natural, female bodybuilders that perform a training load-matched control intervention.

**Fig. 3:**
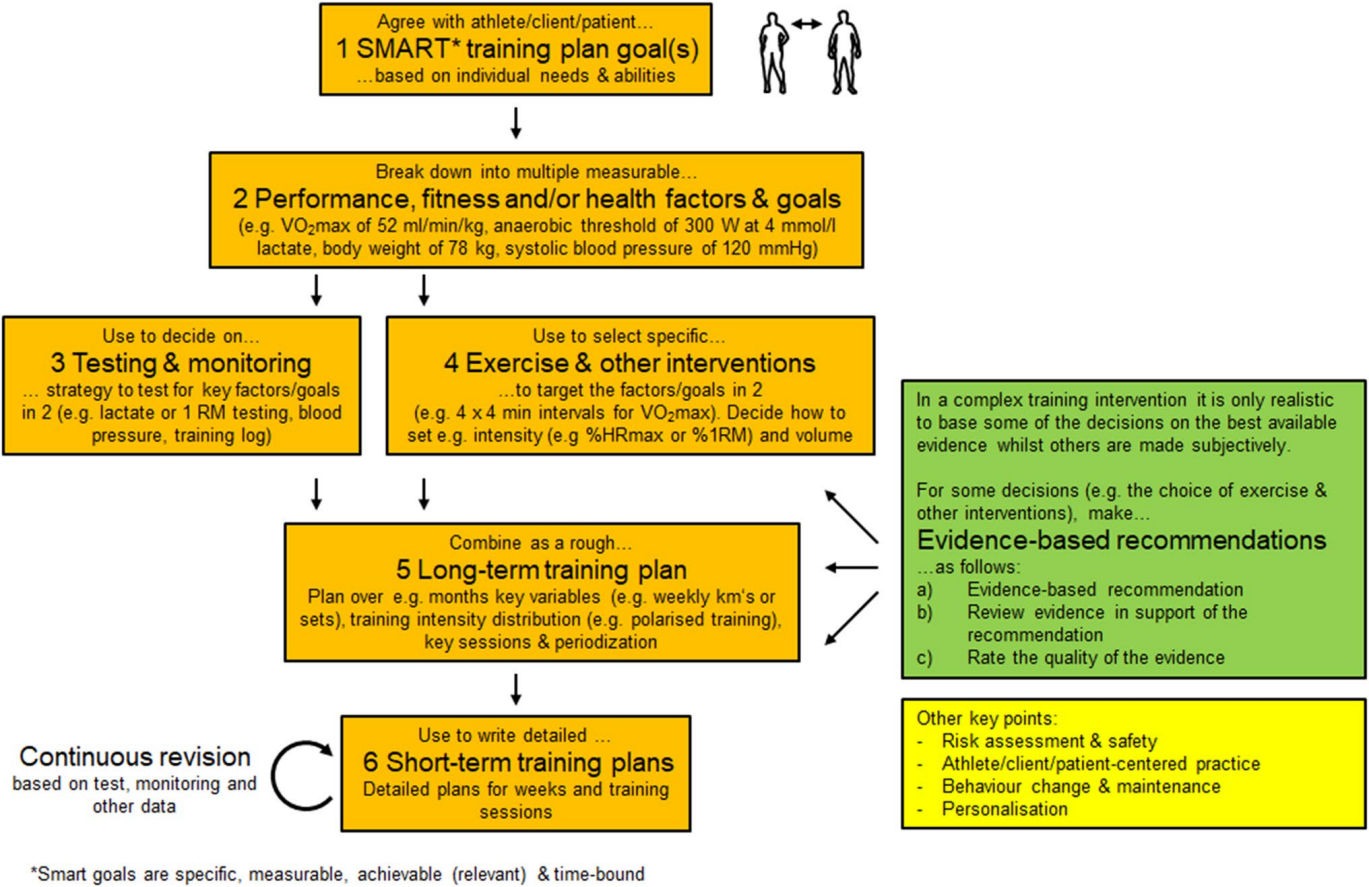
Schematic detailing how an evidence-informed training plan could be written. This approach has six steps. Given the number of decisions that need to be made, it is virtually impossible and not time effective to base all necessary decisions on the best available, scientific evidence. The green box describes a good practice suggestion for making evidence-based recommendations. Other points are listed in a yellow box which are to risk assess the training plan and to make it safe, to consider strategies for behaviour change especially for athletes, clients or patients that are at risk of dropping out, to make the practice athlete/client/patient-centered and to develop a personalization strategy e.g. by checking for responsiveness by monitoring training variables

For this reason, we use the terms evidence-informed training plan or evidence-informed exercise prescription to highlight that only some of the underlying decisions were made with an evidence-based approach, whilst other decisions are based on personal experience. So how can we write an evidence-informed training plan or exercise prescription? Fig. 3 proposes a systematic but subjective strategy to write an evidence-informed training plan for an athlete, client or patient.

Step 1 and 2 define the overall goal and break it down into smaller factors or goals. For example, if the overall goal in step 1 was a sub 3 h marathon in April 2021, then the sub-goals could be a V̇O_2_max of 60 ml/min/kg (subjectively estimated value based on random literature reading), a pace of 4 m/s at 2.5 mmol/L lactate [46] and a BMI of 22 kg/m^2^ [47] at the time of the marathon. For this, earlier competition or test results may be analyzed to ensure that the goals are deemed realistic. Next, in step 3 the practitioner should plan a testing and monitoring strategy. For this, tests should be used that are specific to the desired aims and adaptations and the planned tests should be valid and reliable. Including such testing and monitoring is important because the adaptations of athletes, clients or patients to a training programme can vary greatly. Regular testing and monitoring are not only important in competitive sport but also for interventions that aim to e.g. improve risk factors in patients, as there can be ≈10% adverse responders, where risk factors worsen in response to exercise interventions [20]. If a subject does not sufficiently respond to an intervention, then the dose may be increased [27] or the training plan is altered subjectively. In step 4, training, nutrition and other interventions are chosen to improve the goals stated in step 2 and here, an evidence-based approach is often possible. In step 5, key training variables (e.g. weekly running volume, number of training sessions, longest run and % volume above and below the anaerobic threshold in a marathon runner) can be planned over the entire training plan period and should be agreed with the athlete, client or patient as we typically recommend a subject-centred approach. Based on this systematic process, detailed weekly training plans or individual training sessions are then designed in step 6.

In an evidence-informed approach, some decisions should be based on the best available evidence. How to do this is proposed in the green box in Fig. 3. Evidence-based recommendations can be made for many decisions but are especially useful for the choice of exercise interventions in step 3, as there is often much data on this. After selecting, reading and summarising relevant publications, formulate an evidence-based recommendation clearly and quantitatively e.g. as follows “Train 3 × 30 min of A once weekly over 6 weeks to improve B by x%” (The adaptation “B” is listed in step 2). Second, write a paragraph where you critically describe the scientific evidence in support of your recommendation. Remember, even a single, published training plan by an elite athlete or an expert opinion is evidence, albeit of poor quality. Third, rate the quality of the evidence, e.g. using the GRADE system [9] together with 1 or 2 sentences to explain and justify your rating.

In the yellow box in Fig. 3, we mention four more points: First, a training plan or exercise prescription should not only be effective but must be safe. Therefore, risk assess your training plan and introduce safety measures to mitigate risks. Second, a training plan or exercise prescription may prove useless if an individual disagrees with it or is poorly motivated. Thus, discuss and agree with your training plan with your athlete, client or patient to ensure that it is not against their wishes. Third, even if the individual agrees, he or she may find it difficult to initiate or maintain the exercise behaviours prescribed. Thus, especially with poorly motivated clients or patients, aim to utilise evidence-based behaviour change and maintenance strategies. Fourth, ensure that you have a personalization strategy. Until biomarkers for trainability are identified, practitioners should perform exercise and other tests or monitor training through a training log (step 3) to see whether variables such as the “power at lactate threshold” or “weight lifted” improve as predicted. If an individual does not respond with a sufficient adaptation to training, then the options are to increase the dose of training which has been shown to work for the V̇O_2_max [27], or to try an alternative training method.

Finally, note that the six-step plan is a subjective approach to planning training or prescribing exercise and that there are no studies investigating whether this approach as an intervention works better than alternative approaches.

## Summary and Outlook

In summary, training plans and exercise prescriptions are typically a complex mix of interventions where realistically only some of the underlying decisions can be based on the best, current scientific evidence whilst the remaining decisions are based on unsystematic evidence or experience resulting from practice, academic courses or coaching qualifications.

In this current opinion article, we have suggested a pragmatic six-step strategy detailing how an evidence-informed training plan or exercise prescription could be written. This six-step training plan strategy is a subjective proposal not based on direct research. It also has not been compared to other ways of planning training or prescribing exercise. An added complication is that training responses to a given training programme can vary greatly and hence practitioners need to integrate a personalization strategy to identify non-/poor responders early on. Until biomarkers for trainability are identified, the only way to identify a non-/poor responder is to test and to monitor key training variables to see whether an athlete, client or patient responds to the training intervention. This is, of course, current practice. Moreover, we advocate an athlete, client or patient-centred approach which means following the athlete’s, clients or patient’s wishes even if there are more effective interventions. The trainer’s/coaches’ role is to ensure that the athlete, client or patient can make informed decisions.

Finally, training plans and exercise prescriptions are the points where sport and exercise science knowledge is translated into sport and exercise practice. In our view, methods of how to plan training or prescribe exercise and how to base some of the decisions on scientific evidence should be taught as part of sport and exercise science degrees in the same way as evidence-based, patient-centred and personalized/precision medicine is taught to medical students.
